# Supplementary material for: Red Marine Algae Lithothamnion calcareum Supports Dental Enamel Mineralization
Source: Mar Drugs. 2023 Feb 2;21(2):109. doi: 10.3390/md21020109 (PMC9963885; doi:10.3390/md21020109)
Supplement: Supplementary file 1 [file marinedrugs-21-00109-s001.zip › marinedrugs-2092752-supplementary.pdf]

Appendix A

Supplementary Material

Marcela R. Carrilho, Walter Bretz

Methods

Preliminary Data

LC extract solubilization

After sequential attempts to solubilize the LC (Table S1) extract in different diluents (PBS, distilled water, DMSO, ethanol) the maximum solubilization achieved at physiological pH (6.9) was 0.15g of sieved powder of L.C. in 100mL of DMSO. Using distilled water, the most concentrated solution was obtained by suspending 0.078g of sieved LC in 100 mL and only after titrating it with 1.0 M HCl to pH 2 (a pH incompatible for human applications). Finally, when 0.03g of sieved LC extract was suspended in 100mL, it was possible, after titration with 0.1 M HCl, to get a solution in a physiological pH (6.8) with no need for solution filtration. Then, that solution containing 0.03g LC/100mL of distilled water or 0.03% LC (pH 6.9) was taken as reference for all experiments hereinafter.

Table S1. Total elemental composition of *Lithothamnium calcareum* extract – Vitalidade 50+®

ANALYTE	UNITS	*L. calcareum*	ANALYTE	UNITS	*L. calcareum*
Aluminum	ppm	1420	Mercury	ppm	0.011
Antimony	ppm	4.57	Molybdenum	ppm	<0.1
Arsenic	ppm	0.11	Neodymium	ppm	3.51
Barium	ppm	5.57	Nickel	ppm	0.074
Beryllium	ppm	0.081	Niobium	ppm	<0.1
Bismuth	ppm	2.19	Osmium	ppm	<0.05
Boron	ppm	12.1	Palladium	ppm	<0.05
Bromine	ppm	11.5	Phosphorus	ppm	169.
Cadmium	ppm	<0.03	Platinum	ppm	<0.05
Calcium	ppm	279,000	Potassium	ppm	427.
Carbon	ppm	124,000	Praseodymium	ppm	0.63
Cerium	ppm	2.11	Rhenium	ppm	<0.2
Cesium	ppm	2.66	Rhodium	ppm	<0.05
Chloride	ppm	2370.	Rubidium	ppm	27.6
Chromium	ppm	5.10	Ruthenium	ppm	0.055

Cobalt	ppm	0.107	Samarium	ppm	0.67
Copper	ppm	1.59	Scandium	ppm	0.685
Dysprosium	ppm	1.15	Selenium	ppm	0.060
Erbium	ppm	18.9	Silica	ppm	28,000
Europium	ppm	<0.1	Silver	ppm	1.78
Fluoride	ppm	7.55	Sodium	ppm	3970.
Gadolinium	ppm	3.04	Strontium	ppm	2190.
Gallium	ppm	1.27	Sulfur	ppm	940.
Germanium	ppm	3.08	Tantalum	ppm	<0.05
Gold	ppm	<0.05	Tellurium	ppm	0.066
Hafnium	ppm	<0.1	Terbium	ppm	<0.2
Holmium	ppm	<0.1	Thallium	ppm	0.33
Indium	ppm	0.17	Thorium	ppm	0.081
Iodine	ppm	10.6	Thulium	ppm	<0.05
Iridium	ppm	<0.05	Tin	ppm	0.197
Iron	ppm	10,100.	Titanium	ppm	31.1
Lanthanum	ppm	0.314	Tungsten	ppm	<0.05
Lead	ppm	0.061	Vanadium	ppm	12.9
Lithium	ppm	3.90	Ytterbium	ppm	0.237
Lutetium	ppm	0.310	Yttrium	ppm	1.74
Magnesium	ppm	99,800	Zinc	ppm	16.2
Manganese	ppm	90.5	Zirconium	ppm	0.642

<= no quantities of this analyte detected above the stated limit
Source: 2003 test certificate for Vitalidade 50+®, by Western Analysis, Inc. (Salt Lake City, Utah) for Phoster Algamar (Rio de Janeiro, Brazil).

Calcium, Phosphorus, Magnesium and Fluoride content in solubilized LC extract

Two solutions of LC extract at the concentrations of 0.01% and 0.03% (pH 6.9) were prepared where their Ca, P and Mg contents were evaluated by inductively coupled plasma - atomic emission spectrometry (ICP-OES Radial - Spectro, Arcos, Germany) as described by Sium et al., (2016), while F content was measured by UV-visible absorption analysis (Beckman DU-68, Beckman Coulter, USA) using titration with 5-arylidenebarbituric acid derivatives to detect colorimetric variation [Saravanan et al., 2014]. Operating parameters for ICP-OES are seen in Table S2. Experiments were carried out in triplicate.

Table S2. Operating conditions of the ICP-OES

Parameter	Setting
Power	1.4 kW
Radiofrequency of RF generator	27.12 MHz
Plasma gas glow	12 L min^{-1}
Auxiliary gas flow	1 L min^{-1}
Nebulizer flow	0.85 L min^{-1}
Sample introduction flow	0.85 L min^{-1}
Nebulizer type	Crossflow
Pump speed	30 rpm

Experiments

Experiment 1 – In vitro study - Enamel Blocks Preparation, Artificial demineralization-induction and Hardness measurements after chemical challenging and treatment with LC or NaF

After rinsing in running water for 6 hours and removal of organic debris, calculus and pulp tissue, bovine teeth crowns were separated from roots using a high-speed hand piece under copious water irrigation. From each crown quadrangular teeth fragments were obtained (Isomet, Buehler Ltd. Lake Bluff, IL, USA) using two diamond saws (Extec Corp., Enfield, CT, USA), which were separated by a 4-mm wide spacer, resulting in fragments with average dimensions of 4 x 4 x 2.5 mm.

To check whether demineralization protocols produced caries-like lesions, blocks (n=10) were selected and analyzed for cross-sectional hardness (SHC). All tested specimens where longitudinally sectioned, embedded in acrylic resin and polished. Three rows of 8 indentations each were performed, one in the central region of the exposed dental enamel and the other two 100 mm below and above this, under a 25 g load for 10 s. Indentations were made at 10, 30, 50, 70, 110, 220 and 330 from the outer enamel surface. The mean values at all 3 measuring points at

each distance from the surface were then averaged [Magalhães et al., 2008]. Figure S1 summarizes all possible outcomes for SHC following demineralizing challenge and treatment.

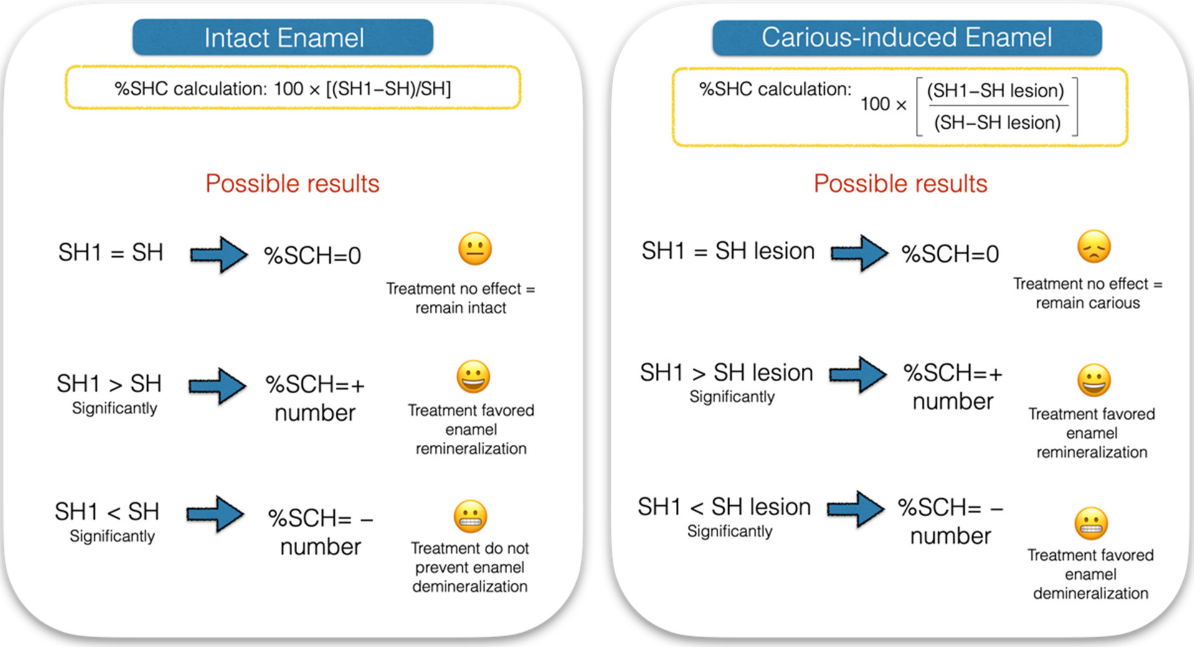

Figure S1. Simulation of possible outcomes as to make %SHC values amenable to interpretation.

Experiment 2 – In situ/ex vivo study

Twins Zygosity Assessment: DNA was extracted from peripheral venous blood samples. DNA marker loci were PCR amplified using standard methods. Amplification products were detected by blood kits (QIAamp, Qiagen Inc., Valencia, CA, USA) with an ABI-377 fluorescent sequencer and analyzed by GENESCAN 2.1 (Applied Biosystems, Foster City, CA, USA). Zygosity was determined by genotyping all individuals for 8 highly polymorphic DNA loci (on chromosomes 2, 7, 11, 17 and 20). Individuals discordant for one or more markers were considered dizygotic.

Enamel Blocks: Specimens (4 x 4 x 2.5 mm) were obtained from the crown of bovine teeth as previously described. Baseline hardness, enamel artificial demineralization-induction and hardness measurements after chemical challenging were also performed as described for the in vitro study.

L. calcareum 0.03%, NaF 0.05% and placebo rinsing preparation: Sterilized water was either used as placebo or to prepare test-rinsing solutions with L. calcareum extract (LC) or sodium fluoride (NaF). A final 0.03% L. calcareum extract solution was prepared and, after titration with 0.1 M HCl, its pH was adjusted to 6.8. The same amount of an 0.05% NaF solution was prepared and adjusted to pH 6.8. The placebo solution was simply the vehicle used to prepare LC or NaF solutions, that is, distilled water adjusted to pH 6.8. Rinses were stored in dark bottles, which were consecutively numbered according to the randomization schedule.

Quantitative light-induced fluorescence (QLF) measurements: QFL is a non-destructive technique that is based on principles of light transmission, absorption, scattering. Imaging of enamel samples were obtained by the QLF system (Inspektor Research Systems BV, Amsterdam, The

Netherlands) which was comprised of a light box containing a xenon bulb and a handpiece, similar in appearance to an intraoral camera. Images were acquired using QLF software (Version 3.03 Inspektor Research Systems BV, Amsterdam, The Netherlands). Once the image of an enamel sample was captured, a quantitative assessment of the demineralization status of the enamel sample was determined. This is undertaken using QLF 2.0 (Inspektor Research Systems BV). Following, the software used the pixel values of the intact enamel to reconstruct the surface of the enamel sample and then subtracts those pixels which are considered to be demineralized.

Images of enamel samples were obtained at baseline (after the lesions were created) and after the *in-situ* phase (i.e., rinsing with LC vs. NaF; LC vs. placebo). All images were obtained under the same conditions. QLF variables include average fluorescence loss (ΔF), size of the lesion (area in mm2), and fluorescence loss integrated over the lesion size (ΔQ = [ΔF x area]). The software calculates these variables using a threshold of difference of 5% from the reconstructed image. Calculations were performed using a 5% threshold. Data examiners were blinded regarding to the treatments being analyzed.

Results

Experiment 1

Element concentrations for both tested solutions in mg/L (≈ ppm) are described in Table S3. Ca and Mg were detected in both LC solutions with higher levels detected in the 0.03% LC solution whereas P and F concentrations were under the instrument detection limits.
After solubilization, the inductively coupled plasma-atomic emission spectrometry (ICP-OES Radial - Spectro, Arcos, Germany) was used to determine the final amount of Ca and Mg in the 0.03% L. calcareum solution which were found to be, respectively, 13.5 mg/L (or 13.5 ppm) and 1.01 mg/L (or 1.01 ppm).

Table S3. Calcium (Ca), Phosphorus (P), Magnesium (Mg) and Fluoride (F) concentrations in testing LC solutions analyzed by inductively coupled plasma - atomic emission spectrometry (ICP-OES) or UV-visible spectrophotometry.

	Elements Concentration (mg/L)			
	**Ca***	**P***	**Mg***	**F****
**0.01% LC**	4.7 ± 0.01	<IDL	0.4 ± 0.01	<IDL
**0.03% LC**	13.5 ± 0.02	<IDL	1.0 ± 0.005	<IDL

*Analyzed by ICP-OES
**Analyzed by UV-visible absorption
<IDL: values below the instrument detection limit

Experiment 2

Assessment of the oral soft tissues was conducted by visual examination of the oral cavity. The structures examined include the gingiva (free and attached), hard and soft palate, oropharynx/uvula, buccal mucosa, tongue, floor of the mouth, labial mucosa, mucobuccal/mucolabial folds, lips, and perioral area. There were no adverse reactions reported by participants while undergoing study protocols.

References

Magalhães, A.C.; Comar, L.P.; Rios, D.; Delbem, A.C.; Buzalaf, M.A. Effect of a 4% titanium tetrafluoride (TiF4) varnish on demineralisation and remineralisation of bovine enamel in vitro. *J Dent* **2008**, *36*, 158-162.

Saravanan, C.; Easwaramoorthi, S.; Wang, L. Colorimetric detection of fluoride ion by 5-arylidenebarbituric acids: dual interaction mode for fluoride ion with single receptor. *Dalton Trans* **2014**, *43*, 5151-5157.

Sium, M.; Kareru, P.; Keriko, J.; Girmay, B.; Medhanie, G.; Debretsion, S. Profile of Trace Elements in Selected Medicinal Plants Used for the Treatment of Diabetes in Eritrea. Scientific World Journal, 2016: 2752836.
